# Active Nerve Regeneration with Failed Target Reinnervation Drives Persistent Neuropathic Pain

**DOI:** 10.1523/ENEURO.0008-17.2017

**Published:** 2017-02-03

**Authors:** Wenrui Xie, Judith A. Strong, Jun-Ming Zhang

**Affiliations:** Pain Research Center, Department of Anesthesiology, University of Cincinnati College of Medicine, Cincinnati, Ohio 45267

**Keywords:** GAP43, regeneration, semaphorin 3A, spared nerve injury, spinal nerve ligation, spontaneous activity

## Abstract

Peripheral nerves can regenerate and, when injured, may cause neuropathic pain. We propose that the active regeneration process plays a pivotal role in the maintenance of neuropathic pain. In one commonly used rodent neuropathic pain model, pronounced pain behaviors follow ligation and cutting of the L5 spinal nerve. We found that the injured nerve regenerates into the sciatic nerve and functionally reinnervates target tissues: the regenerated nerve conducts electrical signals, mechanical responses, and tracers between the leg/hindpaw and axotomized sensory ganglion. The regenerating nerve is the primary source of abnormal spontaneous activity detected *in vivo*. Disrupting the regeneration inhibited pain. First, semaphorin 3A, an inhibitory axonal guidance molecule, reduced functional regeneration, spontaneous activity, and pain behaviors when applied to the injury site *in vivo*. Second, knockdown of the upregulated growth-associated protein 43 (GAP43) with siRNA injected into the axotomized sensory ganglion reduced pain behaviors. We next examined the spared nerve injury model, in which pain behaviors are essentially permanent. The regeneration resulted in tangled GAP43-positive neuromas at the nerve injury site without target reinnervation. Perfusing the nerve stump with semaphorin 3A, but not removing the tangled fibers, prevented or reversed pain behaviors. This effect far outlasted the semaphorin 3A perfusion. Hence, in this model the long-lasting chronic pain may reflect the anatomical inability of regenerating nerves to successfully reinnervate target tissues, resulting in an ongoing futile regeneration process. We propose that specifically targeting the regeneration process may provide effective long-lasting pain relief in patients when functional reinnervation becomes impossible.

## Significance Statement

After peripheral nerve injury, an active regeneration process may play a pivotal role in maintaining neuropathic pain. In two different rat neuropathic pain models, genetic or pharmacological blockade of regeneration reduced pain behaviors. In the spinal nerve injury model, functional regeneration of the ligated spinal nerve was observed, contrary to long-held assumptions. In the long-lasting spared nerve injury model, a tangled neuroma rather than effective target reinnervation was observed. *In vivo* perfusion of the neuroma with semaphorin 3A, an inhibitor of regeneration, also reversed established pain. The persistent neuropathic pain in this model may reflect the anatomical inability of the regenerating nerves to successfully reinnervate target tissues. Specifically targeting the regeneration process may provide long-lasting pain relief.

## Introduction

Neuropathic pain conditions may result from injury to peripheral nerves. This includes conditions such as phantom limb pain, in which a nerve is completely transected; conditions in which a partial nerve injury occurs, such as post-thoracotomy pain; and conditions due to nerve stretch, laceration, or compression. In some cases, relatively minor injuries lead to disproportionately painful conditions, such as complex regional pain syndrome. Despite decades of efforts in understanding the mechanisms of neuropathic pain, it is still not clear why this intractable condition persists far longer than the initial injury.

Unlike the central nervous system, the adult peripheral nervous system is capable of regeneration, and sciatic nerve transection is a rodent model commonly used to study this process (Geuna, 2015). Such studies have elucidated the shift in gene expression in the dorsal root ganglion (DRG) proximal to the injury, from a neurotransmission- to a regeneration-oriented profile, as well as processes in the distal degenerating nerve, including mechanisms by which resident and infiltrating immune cells and reprogrammed Schwann cells modify the distal segment of the injured nerve. The latter processes initially promote regeneration of the nerve into the distal segment along its previous path, but after these processes have continued for some time, the window of opportunity for regeneration closes and the substrate in the distal nerve segment no longer supports regeneration (Mar et al., 2014; DeFrancesco-Lisowitz et al., 2015; Jessen et al., 2015). Thus, peripheral nerve regeneration is less successful when large gaps must be bridged, when the regenerating nerve must grow long distances to reinnervate its targets, or when regeneration is otherwise delayed.

Although sciatic nerve transection was also one of the earliest rodent models of chronic pain, the denervation of the hindlimb makes behavior measurements (other than autotomy) unfeasible, so many neuropathic pain studies use other models involving partial injuries to the sciatic nerve, allowing measurements of pain behavior related to the hindpaws (Jaggi et al., 2011). Most laboratories focus either on pain or peripheral nerve regeneration. Thus, studies of molecular interventions that reduce pain behavior or correlates do not generally consider the possible effects on regeneration, and studies of interventions designed to improve peripheral nerve regeneration do not examine pain behavior. This is despite the fact that numerous molecules (e.g., various cytokines and trophic factors) are implicated in both pain and regeneration (Dubový, 2011).

Our previous studies have focused on pain behavior and mechanisms, using several rat models of neuropathic pain based on injuries to the sciatic nerve (Xie et al., 2005, 2010, 2015). During experiments using the widely used spinal nerve ligation (SNL) model (Kim and Chung, 1992), in which the L5 spinal nerve is ligated and/or cut close to the L5 DRG, we observed that neuronal tracers could be transported from the paw through the transection site to the DRG, contradicting the long-held assumption that the transected spinal nerve does not regenerate and reinnervate the target tissue in this model (Yoon et al., 1996; Hammond et al., 2004; Tandrup, 2004; Djouhri, 2016). This observation led us to investigate the relationship between pain behaviors and the regeneration process. In this article, we present data suggesting a tight link between regeneration and persistent neuropathic pain in two different rat models of neuropathic pain. In addition to the spinal nerve ligation model, we also used the spared nerve injury (SNI) model (Decosterd and Woolf, 2000), in which pain behaviors are essentially permanent. In this model, two of the three branches of the sciatic nerve are ligated and cut at mid-thigh level, with a 2–4 mm gap introduced. This results in long-lasting, profound mechanical allodynia. In addition, the rats avoid weight bearing on the affected foot (guarding behavior), which is considered to be a measure of spontaneous pain (Xu and Brennan, 2010). In the original description of this model (Decosterd and Woolf, 2000), it was proposed that the long duration of the pain behaviors required that the injured axons not reinnervate their peripheral targets. Supporting this idea, they found that a crush injury rather than ligating and cutting resulted in pain behaviors that began to resolve after some weeks. We hypothesized that the long duration of the SNI model might be related to this failure to successfully reinnervate target tissues and hence turn off the regeneration process.

## Materials and Methods

### Animals

The experimental protocol was approved by the University of Cincinnati Institutional Animal Care and Use Committee. Experiments were conducted in accordance with the National Institute of Health *Guide for the Care and Use of Laboratory Animals*. Adult Sprague Dawley rats of both sexes (Harlan) were used. There were no obvious differences observed between males and females in any of the parameters examined, so, except where indicated, data from both sexes have been combined, and experiments were conducted in both males and females in approximately equal numbers.

### Surgical procedure for SNL of the DRG

Rats received a unilateral ligation of the ventral ramus of the L5 spinal nerve based on the description of Kim and Chung (1992) with some modifications. Rats were anesthetized by isoflurane. The skin on the back was cut along the spine from S1 to L4 (DRG level). The L5 and L4 transverse processes were exposed by separating the overlying muscles, after which the ventral ramus of the L5 spinal nerve could be visualized and dissected free from surrounding tissue. After isolation, the L5 spinal nerve was tightly ligated with 6-0 silk ∼2-3 mm distal to the ganglion and then cut ∼1 mm distal to the suture. The incision was closed in layers.

### Perfusion of SNL site

In some experiments, the proximal SNL injury site was perfused with the indicated drugs via tubing connected to an Alzet 14 d osmotic pump with a flow rate of 0.5 µl/h (catalog #2002, Durect), which was implanted just after the SNL ligation and cutting of the spinal nerve was completed. This method was used to apply tetrodotoxin (TTX), to block spontaneous activity, or semaphorin 3A, an inhibitory axon guidance molecule, to block regeneration. The pump was placed underneath the skin along the left side of the spine and ∼5 cm of silicone tubing (Silastic laboratory tubing, catalog #508-002, Dow Corning; inner diameter (i.d.), 0.51 mm; outer diameter (o.d.), 0.94 mm) was led to the injury site. Two cuts were made in the tubing parallel to the long axis and directly opposite each other to divide the final 5 mm of the tubing into two halves, and one-half was removed. The resulting piece of hemisected tubing was gently eased over the proximal cut end of the L5 spinal nerve distal to the suture, and secured in place so that the end of the full-diameter section of the tubing was in close contact with the cut end of the spinal nerve. In order to secure the tubing, the suture ends were left longer than in the usual SNL surgery and were used to tie a second knot securing the hemisected tubing end on the dorsal side of the injury site of the loop of suture surrounding the spinal nerve. The orientation of the tubing was such that the flow direction was in the distal-to-proximal direction along the spinal nerve. The concentration of semaphorin 3A (16 µg/ml) used in the pump was chosen based on pilot experiments showing that a concentration of 8 µg/ml had a smaller effect on pain behaviors than did 16 µg/ml. For experiments perfusing TTX, the concentration was 250 µg/ml, which was based on prior *in vivo* studies (Xie et al., 2005). Drugs were dissolved in artificial CSF (ACSF; in mm: NaCl 130, KCl 3.5, NaH_2_PO_4_ 1.25, NaHCO_3_ 24, dextrose 10, MgCl_2_ 1.2, CaCl_2_ 1.2, and 16 HEPES, pH 7.3, when bubbled with 95% O_2_/5% CO_2_). In control experiments, the site was perfused with ACSF (“vehicle”).

### Procedure for *in vivo* injection of siRNA into the DRG

siRNAs directed against rat growth-associated protein 43 (GAP43) (NCBI gene ID 29423) and nontargeting control were designed by and purchased from Dharmacon/ThermoFisher, as previously described. The siRNA was siGENOME siRNA consisting of a “smartpool” of four different siRNA constructs combined into one reagent. Catalog numbers were M-080112-01 (GAP43) and D-001210-02 (nontargeting control directed against firefly luciferase, screened to have minimal off-target effects and least four mismatches with all known human, mouse, and rat genes according to the manufacturer). The sequences of the GAP43 siRNA were as follows: GAAAGAAGCUGUAGAUGAA, GGAGAUGGCUCUGCUACUA, UGAACAAGAUGGUGUCAAA, and GACAGAAAGUGCUGCUAAA. During the SNL surgery, just prior to ligation of the spinal nerve, 3 µl aliquots containing 80 pmol of siRNA made up with cationic linear polyethylenimine (PEI)-based transfection reagent (in vivo-JetPEI, Polyplus Transfection, distributed by WVR Scientific) were injected into the L5 DRG, through a small glass needle (o.d., 75 µm) inserted close to the DRG through a small hole cut into the overlying membrane close to the site where the dorsal ramus exits the spinal nerve, as previously described (Xie et al., 2012).

### Surgical procedure for SNI

The SNI model was produced as previously described (Decosterd and Woolf, 2000). Briefly, under isoflurane anesthesia, the sciatic nerve and its three terminal branches (the sural, common peroneal, and tibial nerves) were exposed at low-thigh level by blunt dissection through the biceps femoris muscle. The common peroneal and tibial nerves were tightly ligated with 6.0 silk, while the sural nerve was spared. The nerves distal to the ligature were sectioned and 2-4 mm of the nerve stump was removed. Care was taken to avoid any contact with or stretching of the intact sural nerve. Muscle and skin were closed in layers. Each animal was allowed to recover for 24 h before further testing.

### Perfusion of SNI site

In some experiments, the SNI site was perfused with drugs in ACSF or ACSF alone starting at the time of SNI surgery. The method was similar to that used in the SNL model except that the 14 d osmotic pump was embedded underneath the skin covering the thigh, and the tubing was slightly larger (catalog 508-003, Dow Corning; I.D., 0.64 mm; O.D., 1.19 mm), ∼7 cm in length. As for the SNL model, a hemisected tubing end with one side left longer was created and placed over the injury site (covering both ligated nerves), with the longer side of the tubing on the more lateral side of the nerve and secured with the ends of one of the sutures used to ligate the nerve. The orientation of the tubing was such that the flow direction was distal to proximal with respect to the nerve. In some experiments, the SNI injury site was perfused with drugs starting 12 d after the original SNI surgery, as follows: under isoflurane anesthesia, the injury site was re-exposed. The neuroma was cut ∼1 mm distal to the suture and removed. The tubing was installed as described above and secured with suture ends, which were left longer than usual during the first SNI surgery in preparation for the tubing installation.

### Behavior testing

Static mechanical sensitivity was tested by applying a series of von Frey filaments to the heel region of the paws, using the up-and-down method (Chaplan et al., 1994). A cutoff value of 15 g was assigned to animals that did not respond to the highest filament strength used. In order to observe von Frey responses even at early days after surgical denervation, the region tested in the SNL model was centrally located and within the L4 dermatome, while the test site was moved somewhat more laterally after the SNI model to the sural (spared) nerve territory (Bajrović and Sketelj, 1998). A wisp of cotton pulled up from, but still attached to, a cotton swab was stroked mediolaterally across the plantar surface of the hindpaws to score the presence or absence of a brisk withdrawal response to a normally innocuous mechanical stimulus (dynamic tactile allodynia). This stimulus does not evoke a response in normal animals. Cold sensitivity was scored as withdrawal responses to a drop of acetone applied to the ventral surface of the hindpaw. When observed, responses to acetone or light brush strokes consisted of several rapid flicks of the paw and/or licking and shaking of the paw; walking movements were not scored as positive responses. The stimuli for the dynamic tactile allodynia and cold allodynia tests spread over a wider region than the von Frey test and incorporated still innervated regions in both the SNI and SNL models. Spontaneous guarding behavior was scored (Xu and Brennan, 2010) as 0 (no guarding, paw flat on floor), 1 (mild shift of weight away from paw), 2 (unequal weight bearing and some part of the foot not touching the floor), or 3 (foot totally raised or not bearing any weight); these scores were recorded just before each application of the von Frey filament (six observations per paw total) and averaged.

### *In vivo* fiber recording

Rats were anesthetized with 1% pentobarbital sodium (40 mg/kg) and put on a warm blanket to keep the body temperature close to 37°C. Pentobarbital sodium (20 mg/ml) was infused during the entire experiment through a saphenous vein cannula. For recording after the SNL model was implemented, the spinal cord was exposed from vertebrae L5 to T12; and the L5 dorsal root (ipsilateral to the SNL injury site) was located and isolated after removing the dura. Skin edges of this site were used to form a surrounding pool, filled with warm paraffin oil. The distal sciatic nerve was exposed along the femur; the skin edges were used to form a second surrounding pool that was filled with warm paraffin oil. Stimulating electrodes were placed on the exposed sciatic nerve. Microfilament recordings were made from filaments teased from the L5 dorsal root. Size 5 tweezers were used to tear off the perineurium of the dorsal root at the site of recording. A bundle of fibers, which contained ∼10% of the whole dorsal root, was teased from the exposed root and disconnected centrally. This bundle was further separated into microfilaments of approximately equal diameter (30-50 μm). An individual microfilament was placed on a silver wire recording electrode. Waveforms were analyzed using Spike 2 software, version 8.02, Cambridge Electronic Design.

For each microfilament the following characteristics were checked in the following order: spontaneous activity, receptive fields, and compound action potential evoked by stimulating the sciatic nerve:

(1) The microfilament was examined for 3 min to see whether any spontaneous action potentials were present and how many fibers were firing in each strand. The number of fibers with ongoing activity was estimated by the different spike height and frequency of the action potentials. Spike heights were typically 2-10 times the noise level.

(2) We determined whether the recorded ongoing activity represented activity originating from muscle spindles or joint receptors by observing changed firing frequency or pattern when the muscle tone was changed, which was accomplished by gently poking or pressing the lower limb muscle via a glass bar or by slowly pulling and bending the lower leg by gently grabbing the paw with a pair of Adson forceps (1 × 2 teeth tips). Muscle spindle and joint receptor activity was not included in the final count of spontaneously active fibers.

(3) We systematically searched for fibers that could be activated by applying mechanical stimuli to the skin and muscle, which the sciatic nerve innervates. For the paw, a pair of 10 cm Graefe forceps with 1 × 2 serrated tips was used to gently pinch the paw skin to search receptive fields. The receptive fields in skin or muscle in the upper or lower leg were identified using a glass bar with round tip (diameter, 1.5 mm) to gently stroke the skin or press the muscle.

(4) Finally, we applied stimuli via the distal stimulating electrodes to determine how many individual fibers demonstrated activity conducting through the injury site. We did not attempt to characterize the fibers by conduction velocity since the degree of myelination was presumably changing during the course of nerve regeneration, making it impossible to know what class the individual fiber belonged to before the injury.

This entire procedure was repeated until >80% of dorsal root was teased out. In some experiments, the L5 spinal nerve was retransected at the ligation and injury site just before the recordings were made, in order to confirm that observed signals in other experiments were coming from sites distal to the original ligation site. Fiber recordings in normal rats or rats with prior sham SNL surgery were performed in the same way. These recordings confirmed that in the tested receptive fields, stimuli normally activate L5 fibers under a similar recording configuration in normal or sham SNL rats (with or without sham SNL surgery; data not shown) with the exception of the receptive fields along the midline of the spine just below the L5 DRG, where stimuli evoked activity only after SNL. Receptive fields normally innervated by the L5 dorsal ramus were avoided.

Data are presented as the number of fibers per filament; we did not normalize to the total number of fibers as defined by increasing electrical stimulation (Govrin-Lippmann and Devor, 1978) because it was possible that some recorded spontaneously active fibers or immature regenerated fibers could fail to be activated by the stimulating electrode distal to the injury site. Data from normal or sham-operated rats are not presented in the figures because it was evident that filaments in SNL rats even at late time points contained an estimated twofold to threefold smaller number of fibers activated by mechanical stimuli than filaments of similar diameter recorded in normal rats. This meant that it was difficult to accurately apply the same counting methods to the normal rats as were used in the SNL rats.

### Nerve, paw, and DRG microscopy

Animals were first perfused with 0.1 m phosphate buffer until clear fluid was seen, followed by perfusion with 4% paraformaldehyde for 20 min. For immunohistochemistry, paw skin, peripheral nerve, neuroma, or DRG sections were cut at 40 µm on a cryostat after postfixation in 4% paraformaldehyde, 0.1 m phosphate buffer, and 4% sucrose. GAP43 antibody (1:600; catalog #ab16053, Abcam; RRID: AB_443303) was used for staining of the neuroma sections (SNI model) or DRG sections (SNL model). DRG sections stained for GAP43 were costained for the neuronal marker NeuN (1:200; catalog #Ab104224, Abcam; RRID: AB_10711040). In some experiments, a whole-mount DRG preparation (Xie et al., 2010), instead of the sectioned DRG, was used to examine neurons labeled by DiI. In this case, the whole DRG was dissected out and fixed, the sheath was removed, and the DRG was mounted on a slide and covered with antifade mounting medium.

For experiments testing anatomical nerve regeneration, the following two tracer methods were used: (1) in some experiments, 10 µl of the tracer *Fast* DiI oil (5 mg/ml in DMSO; catalog #D3899, Invitrogen), which can be incorporated into cell membranes, was injected into the paw subcutaneously using 31 gauge insulin syringes just after the spinal nerve was ligated. DiI could still be observed in the paw at the later time points when animals were killed to determine whether DiI had been transported back to the ligated L5 DRG. In some of these experiments, DRG sections were colabeled with NF200 antibody (1:100; catalog #ab82259, Abcam; RRID: AB_1658500) or substance P antibody (1:500; catalog #Ab67006, Abcam; RRID: AB_1143173) to determine whether myelinated and small, predominantly unmyelinated nociceptive cells, respectively, could both regenerate. (2) In other experiments, the long-lasting tracer dextran biotin (catalog #D1956, Invitrogen; molecular weight, 10,000; lysine fixable; dissolved in ACSF at 20 mg/ml), which can be taken up by cut axons, was injected into the spinal nerve using a glass pipette with a tip size of ∼25 µm, then the nerve was ligated just distal to the injection site and cut as usual. At the indicated postoperative time points, animals were killed and the paw skin and sciatic nerve sections were examined for biotin-labeled fibers. Dextran biotin in paw skin or sciatic nerve just distal to the knee was visualized by incubating sections overnight in Alexa Fluor 488 streptavidin [diluted 1:500 in double-deionized H_2_O (ddH_2_O); catalog #S32354, Invitrogen] or Alexa Fluor 594 streptavidin (diluted 1:500 in ddH_2_O; catalog #S32356, Invitrogen).

For quantification of GAP43 staining, images from multiple sections of each DRG or neuroma, selected at random, were captured under an Olympus BX61 fluorescent microscope using Slidebook 6.1 imaging acquisition software (Intelligent Imaging Innovation), and the intensity of the signal was normalized by the area measured. For DRG sections, areas containing predominantly neuronal cell bodies and areas containing predominantly axons (i.e., entering the most proximal part of the spinal nerve, proximal to the SNL injury site) were analyzed separately. For quantification of immunohistochemical staining, all image capture and analysis sessions were performed comparing samples from all experimental groups, prepared with the same staining solutions, and then measured using identical display parameters.

### Validation of GAP43 antibody used

In this study, we show that siRNA directed against GAP43 mRNA reduces staining by the GAP43 antibody. This consistency provides evidence for the specificity of these independent methods of investigating GAP43; the four siRNA sequences used had no overlap with the sequence encoding the antigen used for immunization. In addition, we also replicate the GAP43 upregulation in the DRG and peripheral nerves after peripheral nerve transection, as observed in numerous previous studies using a variety of antibodies to GAP43 and several types of peripheral nerve injury (Schreyer and Skene, 1993). GAP43 upregulation in rat DRG after SNL is used as the positive control for this antibody by the manufacturer, along with observation of a band of appropriate size on a Western blot, blockable by excess of the peptide antigen. The antibody has been shown to detect sensory neuron sprouting in several studies, correlating with other independent measures of sprouting (Cho and Cavalli, 2012; Figueroa et al., 2013) .

### *In situ* microscopy

Images of regenerated spinal nerves were obtained after the dissection of perfused rats, using an AmScope 10 megapixel camera inserted into the eyepiece of a dissecting microscope.

### Statistics and data analysis

Sample sizes for all types of experiments were based on our previous experience using these methods. Behavioral time course data were analyzed using two-way repeated-measures ANOVA with Bonferroni’s post-test to determine on which days experimental groups differed. One animal in one behavior experiment died prior to the end of the experiment, and all its data were excluded. No other animals were excluded from analysis. Animals were assigned to experimental groups at random; for experiments comparing a drug with a control, one animal per cage received each treatment. For experiments involving behavioral measurements, the experimenter was blinded to the experimental status starting at the time the experimental groups diverged (e.g., at the time of application of drug vs control). Fiber recording data (average counts from each rat of the numbers of fibers in each filament with spontaneous activity, distal receptive fields, and proximal receptive fields) were examined with ANOVA, with post-tests as indicated to compare selected groups. To examine the effects of drugs applied *in vivo* on fiber recording data, groups with drug application were compared with groups with vehicle application at the same time points. Differences in GAP43 staining from sections of DRG were analyzed with ANOVA with Dunnett’s post-test comparing the nontargeting siRNA group to normal and to GAP43 siRNA groups. Differences in GAP43 staining from neuroma sections were analyzed with ANOVA with Tukey’s post-test comparing the normal, vehicle-treated, and semaphorin 3A-treated groups. The statistical test used in each case is indicated in the text or figure legend. Two-sided tests were used throughout. Significance was ascribed for *p* < 0.05. Levels of significance are indicated by the number of symbols, as follows: *p = 0.01 to <0.05; **p = 0.001 to 0.01; ***p < 0.001. Data are presented as the average ± SEM.

## Results

### In the SNL model, the ligated and transected L5 nerve can regenerate into the original sciatic nerve

**Figure 1.**
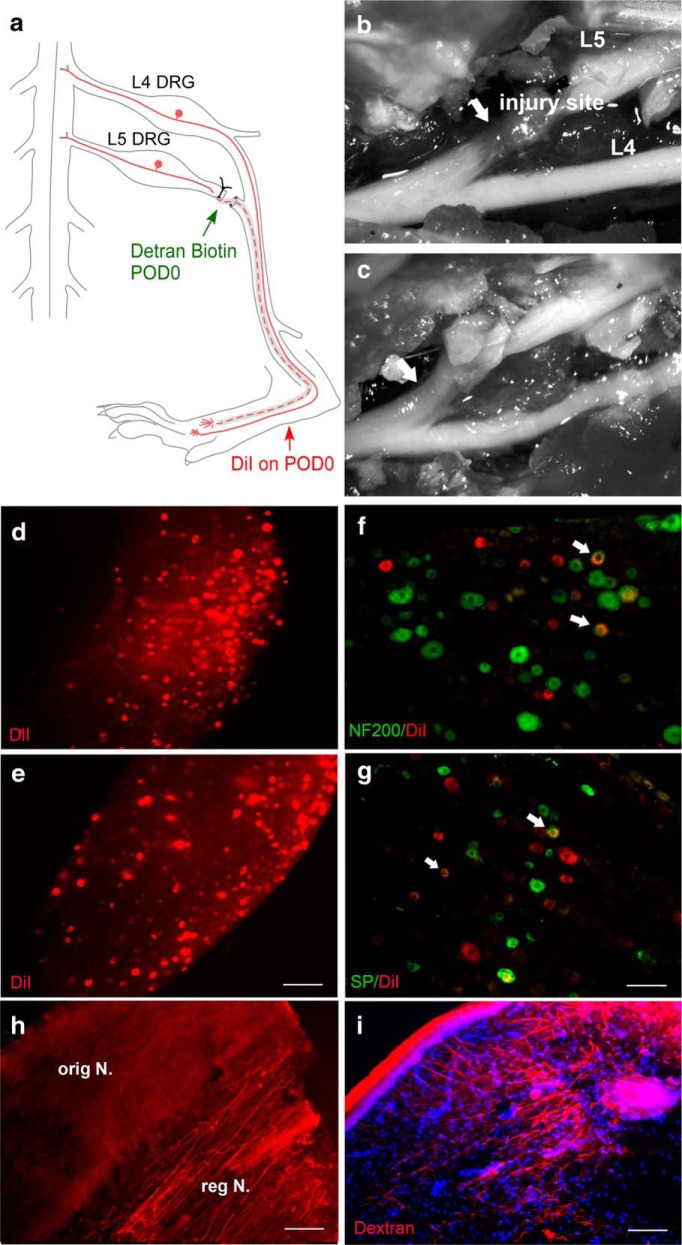
Anatomical evidence that the L5 spinal nerve regenerates in the SNL model. ***a***, Schematic of the SNL model showing the site of L5 spinal nerve ligation and transection distal to L5 DRG and proximal to its merger with the L4 spinal nerve to form the sciatic nerve. The dotted line indicates the regenerated nerve segment. In separate experiments, the tracer dextran biotin (injected into the cut end of the spinal nerve) or DiI (injected into the paw) was injected just after the spinal nerve transection. ***b***, ***c***, *In situ* dissecting microscope images showing the regenerated segment of the L5 spinal nerve (arrows) just distal to the injury sites observed on day 20 (***b***) and day 70 (***c***). ***d***, ***e***, Whole-mount images of the L5 DRG showing DiI transported from the hindpaw (red) on day 20 (***d***; observed in only one of four rats) and day 70 (***e***; observed in six of six rats). ***f***, ***g***, DRG sections showing colocalization (yellow; arrows) of DiI (red) with labeling for NF200 (***f***; green; marker for myelinated cells; repeated in five rats) or Substance P (SP; ***g***; green, marker for nociceptive cells; repeated in five rats). Sections were obtained 70 d after spinal nerve ligation and transection. DiI could still be observed at the paw injection site at this time. ***h***, Longitudinal section of sciatic nerve distal to the original transection site, just below the knee, showing dextran biotin-positive fibers 70 d after spinal nerve transection (repeated in four rats). The regenerating, dextran biotin-positive nerve (“reg N.”) appeared somewhat segregated from the dextran biotin-negative original intact nerve (“orig N.) ***i***, Cross-section of paw skin, showing dextran biotin-positive fibers 35 d after spinal nerve transection (repeated in five rats). Scale bars: ***d***, ***e***, 250 µm; ***f–i***, 100 µm.

In the SNL model, the L5 spinal nerve is ligated and cut proximal to the sites where it merges with the L4 and L6 spinal nerve to form the sciatic nerve (Fig. 1*a*). This is a commonly used model of neuropathic pain, and it is widely assumed that the nerve does not regenerate and/or reinnervate and that therefore evoked pain behaviors are mediated only by the intact neurons in the L4 DRG. While using this model, we observed that the neuronal tracer DiI, injected into the ipsilateral hindpaw just after the spinal nerve transection, could later be observed in the cell bodies of the axotomized L5 DRG. In one of four rats examined, DiI could be observed as early as 3 weeks after the spinal nerve ligation, and in every rat examined at later time points (Fig. 1*d*,*e*). Further investigation using double-labeling showed that DiI could be observed in both NF200-positive neurons (a marker for myelinated neurons; Fig. 1f) and substance P-positive neurons (a marker primarily of unmyelinated nociceptors; Fig. 1*g*). When the long-lasting tracer dextran biotin was injected into the proximal cut end of the spinal nerve at the time of ligation and cutting, dextran biotin-labeled fibers could be observed in the distal sciatic nerve (Fig. 1*h*) and in the hindpaw skin as early as 3 weeks postoperatively (Fig. 1*i*; and see below). Dissection and *in situ* examination of the injury site as early as 2 weeks after the initial surgery showed that the nerve had regenerated from the ventral side of the ligature, just proximal to the suture, and rejoined the sciatic nerve along with the intact L4 spinal nerve (Fig. 1*b*,*c*). At early time points, this newly grown nerve was very translucent (Fig. 1*b*), which may account for the failure to notice it during 10 years of using this model. At later time points, the newly grown nerve appeared whiter, more similar to normal nerve (Fig. 1*c*). The regenerated nerve was observed *in situ* in rats of both sexes, at time points ranging from 2 to 10 weeks after the spinal nerve ligation surgery (24 males and 12 females with spinal nerve ligation, plus 12 males with spinal nerve ligation plus vehicle perfusion of the injury site; see below).

### The regenerated nerve is functional

**Figure 2.**
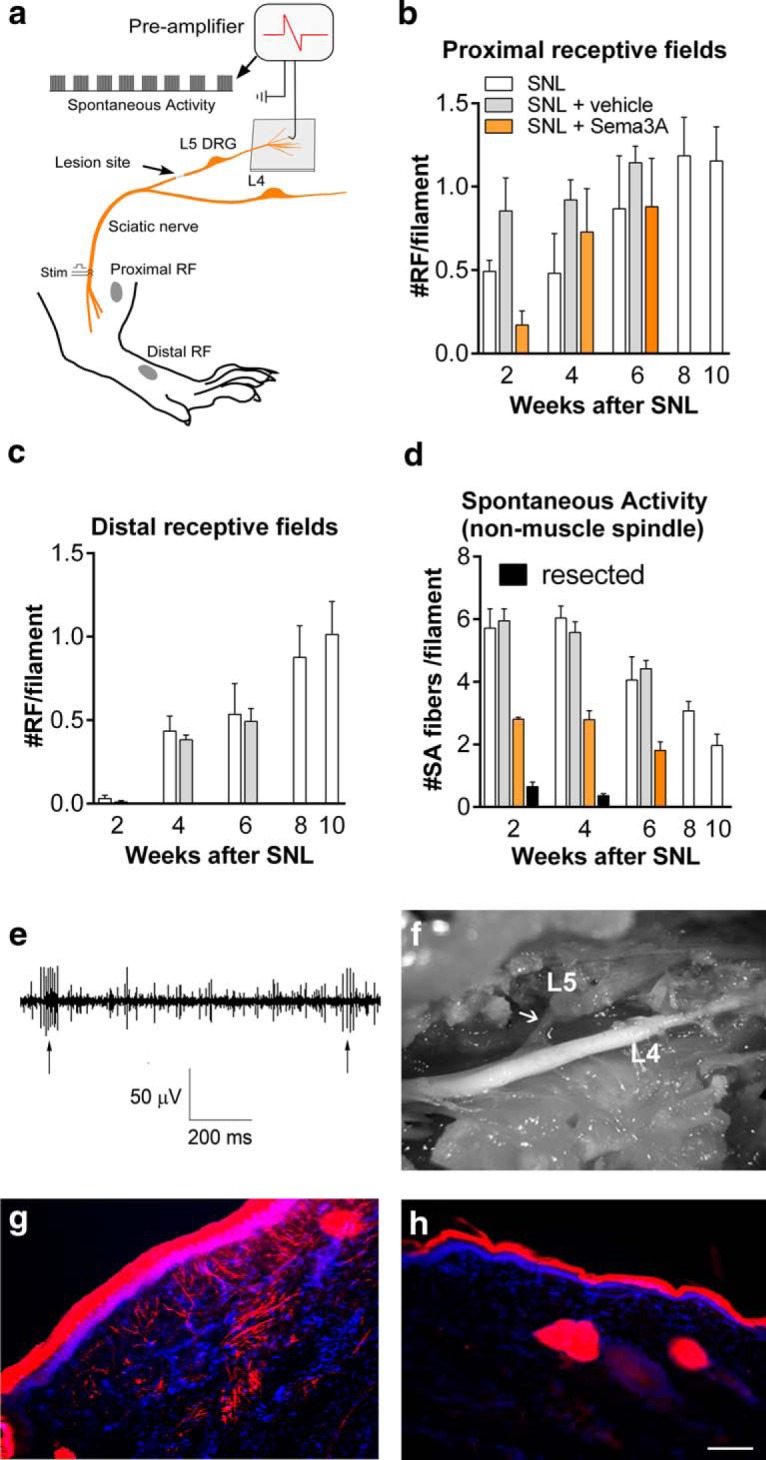
Regeneration of L5 spinal nerve is functional and can be reduced by semaphorin 3A. ***a***, Schematic of the *in vivo* fiber recording setup. Filaments were teased out of the dorsal root, disconnected centrally. After observing spontaneous activity, receptive fields were examined by applying mechanical stimuli to proximal (above the knee) and distal (lower leg and paw) regions. ***b***, ***c***, Average number of proximal (***b***) and distal (***c***) receptive fields per filament detected at the indicated times after SNL, SNL plus perfusion of the injury site with vehicle, or SNL plus perfusion of the injury site with semaphorin 3A (“Sema3A”). In ***c***, values for semaphorin 3A group are zero. ***d***, Average number of spontaneously active fibers per filament (excluding normal muscle spindle and joint receptor activity) detected in the same experiments. *N* = 3 or 4 rats/group with 72 to 116 filaments per group, except for the resected POD14 group, which contained only two animals. ***e***, Example of spontaneous activity, and activity evoked by mechanical stimulation (Stim) of a receptive field (RF; arrows). ***f***, *In situ* image of regenerated nerve on day 28 in animal perfused with semaphorin 3A (tubing removed before image was taken); note that the segment is thin and fine (compare Fig. 1*b*; observation was repeated in eight rats). ***g***, Paw cross-section showing dextran biotin-labeled fibers (red) observed 28 d after SNL in a vehicle-perfused rat (repeated in four rats). ***h***, Dextran biotin-labeled fibers were absent in paw cross-sections obtained from semaphorin 3A-perfused rat (repeated in four rats). Scale bar, 100 µm.

In order to determine whether the regenerated L5 spinal nerve was functional, we used *in vivo* microfilament recording to determine whether stimuli from peripheral receptive fields or electrical stimulation could be conducted through the newly regenerated nerve. The recording electrode was placed on a 30- to 50-μm-diameter filament teased out from the L5 dorsal root and disconnected centrally. A bipolar stimulating electrode was placed on the sciatic nerve distal to the injury site (Fig. 2*a*). Data are presented as the average number of fibers per filament. We first recorded any spontaneously active fibers. Ongoing joint receptor or muscle spindle activity (identified based on the ability to change the activity by manipulating a muscle or a joint) was not counted as spontaneous. After recording spontaneous activity and identifying joint receptor and muscle spindle activity, mechanical stimuli (pressure applied with a blunt glass rod or forceps) were applied to several defined proximal and distal regions normally innervated by L5 to determine whether any of the axons in the filament had receptive fields in those regions (Fig. 2*e*). For analysis purposes, the data were combined into distal fields (muscle spindles, joint receptors, or mechanically sensitive fibers with responses from lower leg, ankle, or heel; Fig. 2*c*) and proximal fields (responses to stimuli detected in upper leg muscle, and muscle or skin along the spine near the tail or on top of the spine near L5; Fig. 2*b*). After testing for mechanical receptive fields, electrical stimuli were applied to the sciatic nerve distal to the injury site to determine whether stimulated action potentials could conduct through the new nerve. Two weeks after the spinal nerve ligation, at least one electrically stimulated action potential could be observed in 17% of filaments. This increased to 86% by week 4, and to 90–95% for weeks 6–10. Fibers with proximal receptive fields could be recorded at week 2, with slightly increasing numbers over time (Fig. 2*b*). Distal receptive fields recovered more slowly; a few fibers with distal receptive fields could be observed at 2 weeks, but these were not robustly observed until week 4 (Fig. 2*c*).

No fibers could be activated by stimulation of any of the receptive fields or muscle spindles or joint receptors, and no action potentials could be stimulated by electrical stimulation of the distal sciatic nerve, when the spinal nerve and any newly grown nerve were recut at the original cut site just prior to the start of the recording session (in 55 filaments recorded from two rats at week 2, and 116 filaments recorded from three rats at week 4).

### *In vivo* application of semaphorin 3A to the injury site reduced functional and anatomical measures of regeneration

In order to pharmacologically manipulate the regeneration process, we developed a method to perfuse the SNL injury site *in vivo*. Fine silicon tubing, open on one side, was placed over the end of the ligated spinal nerve and secured at the same suture used to ligate the nerve. The tubing was attached to an osmotic pump to apply drugs for the first 2 weeks after the SNL surgery. In rats perfused with vehicle, the spontaneous activity and number of receptive fields were generally similar to those observed in rats without pumps (Fig. 2*b–d*). *In situ*, the regenerating nerve was observed to grow out of the ventral side of the ligature; the tubing was secured over the dorsal side and did not appear to affect this process. We used this method to apply semaphorin 3A, an inhibitory axonal guidance molecule. Semaphorin 3A can collapse growth cones in a subset of DRG neurons (Reza et al., 1999) and can also regulate axonal transport, even in neurons that do not show the growth cone collapse response (Goshima et al., 1999). Its receptor is expressed in both normal and axotomized adult DRG (Pasterkamp et al., 1998). After treatment with semaphorin 3A for the first 2 weeks after injury, fibers with distal receptive fields could never be found at any subsequent time point (Fig. 2*c*; *n* = 0 fibers with distal receptive field of 105 fibers in three rats at week 2; 0 of 72 fibers in three rats at week 4; and 0 of 87 fibers from three rats at week 6). The effect of semaphorin 3A on distal receptive fields compared with vehicle was significant (ANOVA, *F*_(5,15)_ = 34.67, *p* < 0.0001, *n* = 3-4/group). In contrast with the distal regions, receptive fields in the proximal regions could be detected in some fibers, however, the recovery after SNL was significantly delayed by semaphorin 3A compared with the vehicle group (ANOVA, *F*_(5,15)_ = 3.07, *p* = 0.04, *n* = 3-4/group), with the largest reduction observed at the 2 week time point (Fig. 2*b*). *In vivo*, a regrowing nerve could be observed near the injury site in semaphorin 3A-treated rats at weeks 2 through 7, but qualitative observations showed that it was much thinner than that observed in rats at the same time points without drug application (Fig. 2*f*). Two week semaphorin 3A application also blocked regeneration of nerve fibers into the paw, as visualized with dextran biotin at week 4 (Fig. 2*g*, vehicle perfusion, *h*, semaphorin 3A perfusion), which is consistent with the observed lack of distal receptive fields.

### The regenerating nerve is the main source of abnormal spontaneous activity after nerve injury

During the fiber-recording experiments, spontaneously active fibers (excluding ongoing activity from muscle spindles and joint receptors) were readily recorded from rats after SNL or SNL with vehicle perfusion of the injury site for the first 2 weeks (Fig. 2*d*). The incidence decreased gradually over time and was significantly lower (*p* < 0.01) at weeks 6–10 compared with week 2 (ANOVA with Dunnett’s post-test comparing week 2 to all other time points in the SNL group, *F*_(4,15)_ = 11.44, *p* = 0.0002, *n* = 4 rats/group). When the nerve stump and newly regenerated nerve at the original injury site were freed from the surrounding tissue and then resectioned just prior to recording, almost all the spontaneous activity was eliminated (Fig. 2*d*; tested at weeks 2 and 4; ANOVA, *F*_(3,9)_ = 42.13, *p* < 0.0001, *n* = 2–4 rats/group, with Bonferroni’s post-test comparing resected to nonresected groups at each time point). Applying semaphorin 3A to the injury site for the first 2 weeks also significantly reduced spontaneous activity at all time points tested (weeks 2, 4, and 6; ANOVA, *F*_(5,15)_ = 29.68, *p* < 0.0001, *n* = 3 or 4 rats/group, with Bonferroni’s post-test comparing vehicle to semaphorin 3A-treated groups at each time point; Fig. 2*d*).

We also examined the effect of blocking spontaneous activity originating from the injury site by perfusing with TTX for the first 2 weeks after surgery. Fiber recordings were conducted at the 4 week time point (i.e., 2 weeks after the end of the TTX perfusion period). TTX perfusion, like semaphorin 3A perfusion, reduced the incidence of spontaneous activity measured at 4 weeks (number of spontaneously active fibers per filament: 2.0 ± 0.2 for TTX; 2.8 ± 0.3 for semaphorin 3A; vs 5.6 ± 0.3 for vehicle perfusion; ANOVA, *F*_(2,8)_ = 44.99, *p* < 0.0001, with Bonferroni’s post-test comparing all groups with each other; both the TTX and semaphorin 3A groups were significantly different from vehicle, *p* < 0.001, *n* = 3 or 4 rats/group). TTX also had effects on the recovery of proximal and distal receptive fields that were similar to those of semaphorin 3A measured at the 4 week time point. TTX, when perfused for the first 2 weeks, prevented recovery of distal receptive fields measured at week 4 (zero distal receptive fields per filament after TTX perfusion, zero after semaphorin 3A perfusion, vs 0.38 ± 0.03 after vehicle perfusion; the vehicle group was significantly different from the other two groups, *p* < 0.001; ANOVA, *F*_(2,8)_ = 156.4, with Bonferroni’s post-test comparing each group to every other group, *n* = 3 or 4 rats/group), while the number of proximal receptive fields observed on week 4 was not significantly different than that observed with vehicle or semaphorin 3A perfusion (0.71 ± 0.13, 0.72 ± 0.3, and 0.92 ± 0.12 proximal receptive fields per filament, respectively, for TTX, semaphorin 3A, and vehicle perfusion; ANOVA, *F*_(2,8)_ = 0.38, *p* = 0.61, *n* = 3 or 4 rats/group).

### Blocking regeneration reduces pain behaviors in the SNL model

**Figure 3.**
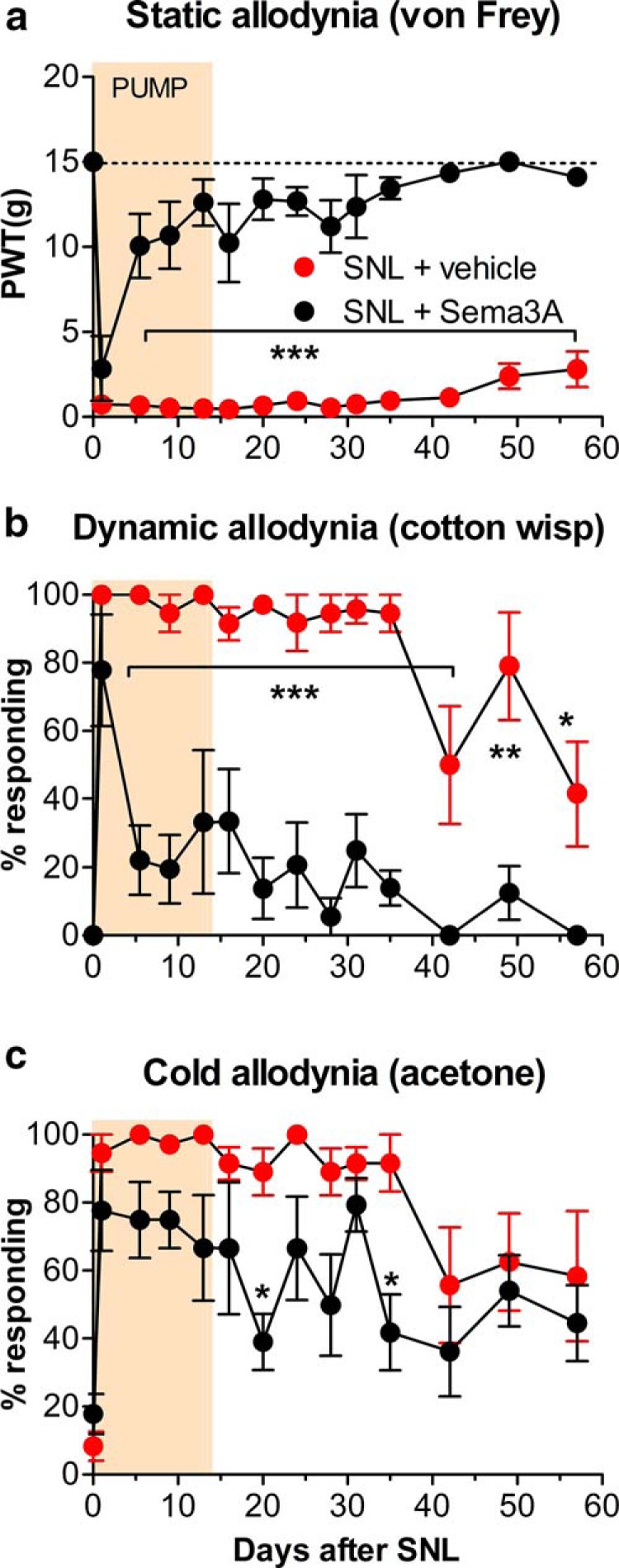
Local perfusion of the injury site *in vivo* with semaphorin 3A reduces pain behaviors in the SNL model. Baseline behaviors (average of two measurements) are plotted on day 0. Immediately after the ligation and transection of the L5 spinal nerve, fine tubing was led from a 14 d osmotic pump to the cut end of the spinal nerve to deliver either vehicle or semaphorin 3A (16 µg/ml), as indicated by the shaded bar. ***a***, Threshold for withdrawal responses to von Frey filaments (*F*_(1,10)_ = 137.58). ***b***, Dynamic allodynia (withdrawal responses to light stroking with a fine cotton wisp; *F*_(1,10)_ = 91.08). ***c***, Cold allodynia (withdrawal responses to a drop of acetone; *F*_(1,10)_ = 13.77). *p < 0.05; **p < 0.01; ***p < 0.001, significant difference between the two groups at the indicated time points, two-way repeated-measures ANOVA, with Bonferroni’s post-test). *n* = 6 male rats/group. PWT, paw withdrawal threshold.

The SNL model leads to pronounced ipsilateral mechanical hypersensitivity (static allodynia) and tactile allodynia (light stroke-evoked dynamic allodynia), as well as cold allodynia, as measured in the ipsilateral hindpaw. We used the *in vivo* perfusion method to measure the effects of reducing regeneration with semaphorin 3A on these pain behaviors. As in the above experiments, the perfusion of the injury site with a 14 d osmotic pump started immediately after the spinal nerve ligation and transection. As shown in Figure 3, *a* and *b*, semaphorin 3A perfusion markedly reduced mechanical hypersensitivity and mechanical allodynia. The reduction was not significant on postoperative day 1 (POD1) but was significant from day 5 through the end of the experiment (8 weeks; i.e., long outlasting the duration of drug application). The effect on cold allodynia (Fig. 3*c*) was much smaller and did not reach significance on most days.

On the contralateral side, SNL induced only minor static allodynia, with von Frey thresholds >12 g on all days tested, and no effect of semaphorin 3A was observed. Contralateral responses to stroking with a cotton wisp during the dynamic allodynia test were never observed in either group. Contralateral responses to acetone did not differ significantly from baseline on any day tested, in either group, and were not significantly affected by semaphorin 3A on most days.

**Figure 4.**
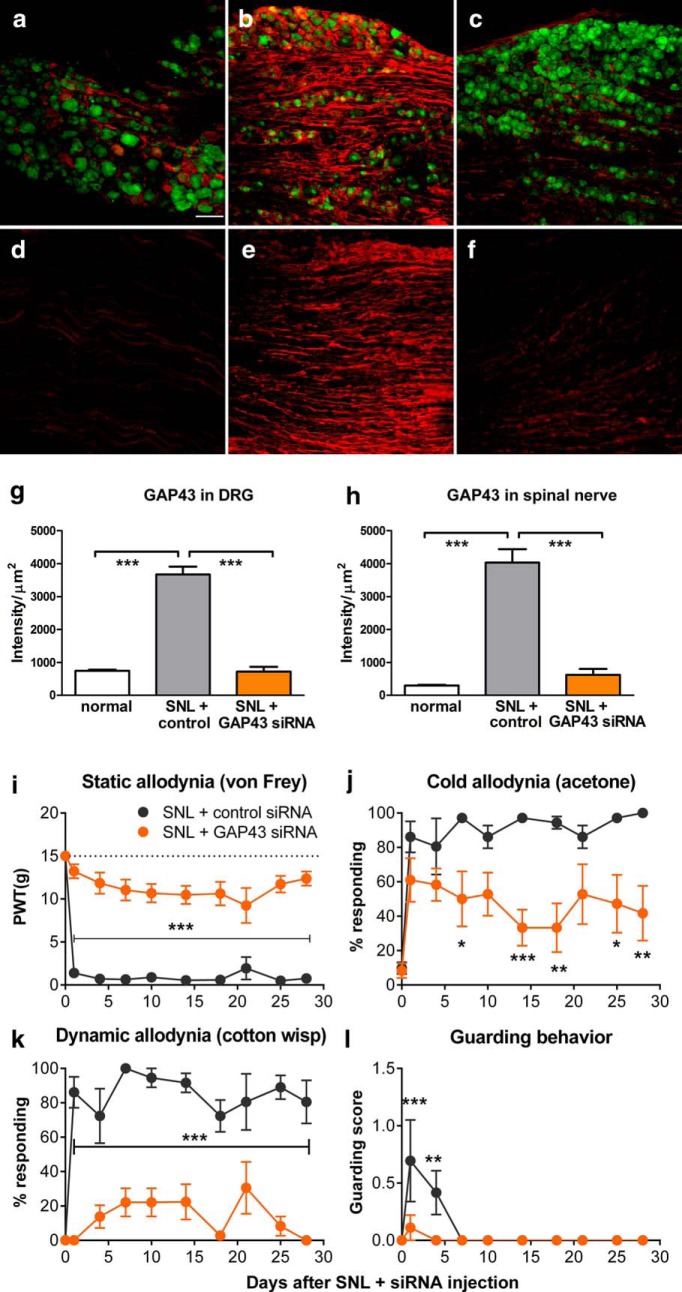
Knockdown of GAP43 in the L5 dorsal root ganglion reduces pain behaviors and GAP43 upregulation in the SNL model. siRNA directed against GAP43 or control nontargeting siRNA was injected into the L5 DRG immediately prior to implementing the SNL model. ***a–f***, Sections were obtained 4 d later. DRG sections from cellular regions (***a–c***) or axonal regions (***d–f***) stained for GAP43 (red) and neuronal marker NeuN (green) from normal (***a***, ***d***), SNL + control siRNA-injected (***b***, ***e***) and SNL + GAP43-siRNA injected (***c***, ***f***) DRGs. ***g***, ***h***, Summary data of GAP43 intensity from cellular (***g***) and axonal (***h***) regions. ***p < 0.001, significant differences between control siRNA groups and both other groups; ANOVA with Dunnett’s post-test. ***g***, *F*_(2,12)_ =107.70; ***h***, *F*_(2,12)_ = 62.77; *n* = 5 rats/group (3 males, 2 females), with 19–33 soma region sections per rat and 6–12 axonal regions per rat. Effect of siRNA injection on pain behaviors: ***i***, Threshold for withdrawal responses to von Frey filaments (dotted line indicates baseline; *F*_(1,10)_ = 119.37). ***j***, Cold allodynia (withdrawal responses to a drop of acetone; *F*_(1,10)_ = 10.77). ***k***, Dynamic allodynia (withdrawal responses to light stroking with a fine cotton wisp; *F*_(1,10)_ = 124.59). ***l***, Guarding behavior score (maximum value is 3; *F*_(1,10)_ = 4.94). *p < 0.05; **p < 0.01; ***p < 0.001, significant difference between the two groups at the indicated time points (two-way repeated measures ANOVA with Bonferroni’s post-test). *N* = 6 rats/group (3 males, 3 female). PWT, paw withdrawal threshold.

Semaphorin 3A can also have effects on immune cells. We therefore used DRG injection of siRNA directed against GAP43, another molecule important for regeneration, as a second, independent method to interrupt regeneration. siRNA-mediated knockdown of GAP43 significantly reduced the upregulation of GAP43 in the DRG. Immunohistochemical staining of DRG sections was performed 4 d after spinal nerve ligation (Fig. 4*a–h*). We observed that the signal for GAP43 was relatively low in normal DRG (Fig. 4*a*). After SNL (and injection of control siRNA), marked upregulation could be observed (measured on POD4; Fig. 4*b*). In the cellular regions of the DRG, the GAP43 signal was especially strong in the axonal processes coursing between the cell bodies (overall density measurements showing approximately fivefold upregulation after SNL in cellular regions in animals injected with control siRNA). In addition, strong staining was also observed in axonal regions just distal to the cellular region and proximal to the SNL lesion site (Fig. 4*e*; ∼13-fold upregulation when compared with the lower initial level in the corresponding axonal regions of normal animals; Fig. 4*d*). In both cellular and axonal regions, the siRNA directed against GAP43 reduced the staining approximately fivefold (Fig. 4*c*) to sixfold (Fig. 4*f*; for summary data, see Fig. *g*,*h*).

As shown in Figure 4*i–l*, GAP43 siRNA injection at the time of spinal nerve ligation also markedly reduced pain behaviors, especially mechanical pain behaviors, which were significantly reduced on all days tested. Similar to the results obtained with semaphorin 3A, the effects on cold allodynia were more modest. Guarding behavior, a measure of spontaneous pain (Xu and Brennan, 2010), was also reduced by the knockdown (Fig. 4*l*).

As in the semaphorin 3A experiments, there were no effects of GAP43 siRNA on contralateral pain behaviors, which were always minimal in both groups (data not shown). Contralateral guarding behavior was never observed in either group.

### Robust regeneration with failed reinnervation in the long-lasting spared nerve injury pain model

**Figure 5.**
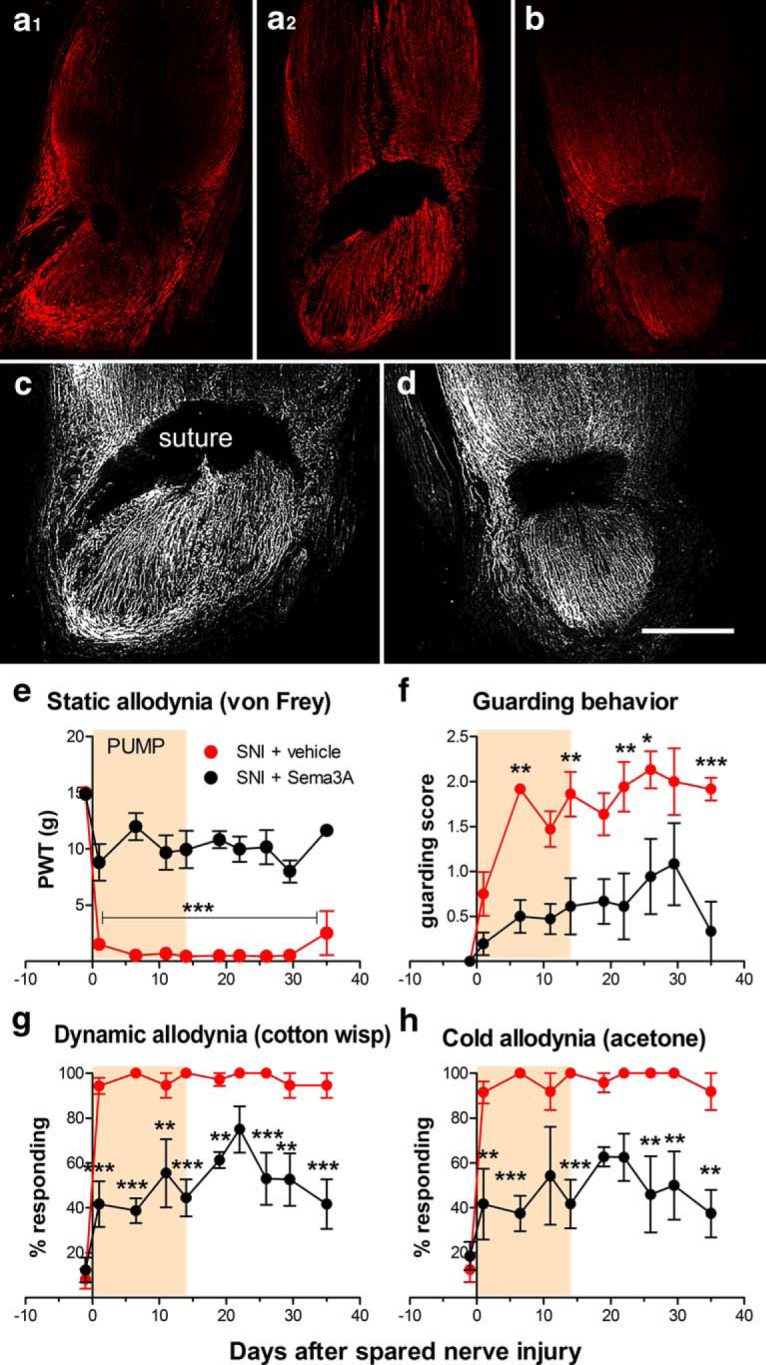
Semaphorin 3A applied to the injury site reduces regeneration and pain behavior in the SNI model. ***a1–b***, Longitudinal sections of the neuroma region at the spared nerve injury site taken 28 d after nerve injury, stained for regeneration marker GAP43 (red). ***a1–b***, The injury site was locally perfused with vehicle (***a1***, ***a2***) or semaphorin 3A (16 µg/ml; ***b***) for the first 14 d after the injury. ***a1***, ***a2***, The same nerve, taken at different depths. The suture can be seen as the two dark regions in ***a1*** and the larger dark region in ***a2*** and ***b***. ***c***, ***d***, Grayscale rendering of portion of the images in ***a2*** and ***b*** with sharpness enhancement to emphasize fiber structure (and to further increase the signal from ***b***). Scale bar, 500 µm. ***e–h***, Effect of perfusing the injury site in the spared nerve injury model with vehicle or semaphorin 3A for the first 14 d after injury on pain behaviors. ***e***, Threshold for withdrawal responses to von Frey filaments (*F*_(1,10)_ = 120.19). ***f***, Guarding behavior score (maximum value is 3; *F*_(1,10)_ = 31.33). ***g***, Dynamic allodynia (withdrawal responses to light stroking with a fine cotton wisp; *F*_(1,10)_ = 70.30). ***h***, Cold allodynia (withdrawal responses to a drop of acetone; *F*_(1,10)_ = 47.98). *p < 0.05; **p < 0.01; ***p < 0.001, significant difference between the two groups at the indicated time points (two-way repeated-measures ANOVA with Bonferroni’s post-test). *N* = 6 rats/group (4 males, 2 female). PWT, paw withdrawal threshold.

The above data suggested a close relationship among nerve regeneration, spontaneous activity in regenerating sensory nerves, and mechanical pain behaviors. We next examined the effects of semaphorin 3A perfusion in the spared nerve injury model. Pain is essentially permanent in this model, and its originators proposed that this might be because target reinnervation was not possible (Decosterd and Woolf, 2000). Based on our results in the SNL model, continuation of this (albeit futile) regeneration process might be a cause of prolonged pain behaviors. As evidence supporting this proposal, we observed that, although injured nerves were able to regenerate, they failed to reinnervate target tissues. The tangled regenerating fibers (visualized by staining for the regeneration marker GAP43) formed a neuroma at the injury site (Fig. 5*a1*,*a2*,*c*), in contrast with the regenerated nerve observed in the SNL model. Perfusion of the injury site *in vivo* with semaphorin 3A, using a method similar to that used for the SNL model, markedly reduced mechanical allodynia (Fig. 5*e*,*g*) and cold allodynia (Fig. 5*h*) as well as guarding behavior (Fig. 5*f*). The behavioral effects of semaphorin 3A long outlasted the 2 week period of drug application. This suggested that the regeneration program was effectively shut down by the semaphorin 3A treatment and did not resume after the treatment ended.

Semaphorin 3A application beginning at the time of SNI surgery greatly reduced regeneration and neuroma formation. Longitudinal sections showed well organized, nontangled GAP43-positive nerve fibers distal to the ligature (Fig. 5*b*,*d*). Semaphorin 3A also reduced the intensity of the nerve regeneration marker GAP43 staining at the injury site and as observed 4 weeks postinjury (i.e., 2 weeks after the end of the drug perfusion period; Fig. 5*b*,*d*). Quantification of the intensity of GAP43 staining in sections of neuroma taken from the injury site 4 weeks after SNI showed a marked (∼75-fold) upregulation of GAP43 in the SNI plus vehicle perfusion group compared with sections taken from the corresponding region of normal sciatic nerve; this was over threefold less in the SNI plus semaphorin 3A perfusion group [46 ± 3.5 mean intensity/µm^2^ in normal rats; 3447 ± 313 mean intensity/µm^2^ in SNI plus vehicle rats; and 1047 ± 119 mean intensity/µm^2^ in SNI plus semaphorin 3A rats; each group differed significantly from every other group; ANOVA with Tukey’s post-test, *F*_(9,11)_ = 81.23, *n* = 4 (2 male and 2 female rats/group) with 10–14 sections per animal].

### Disrupting the regeneration process but not resection of the neuroma reduced already established pain behaviors in the SNI model

**Figure 6.**
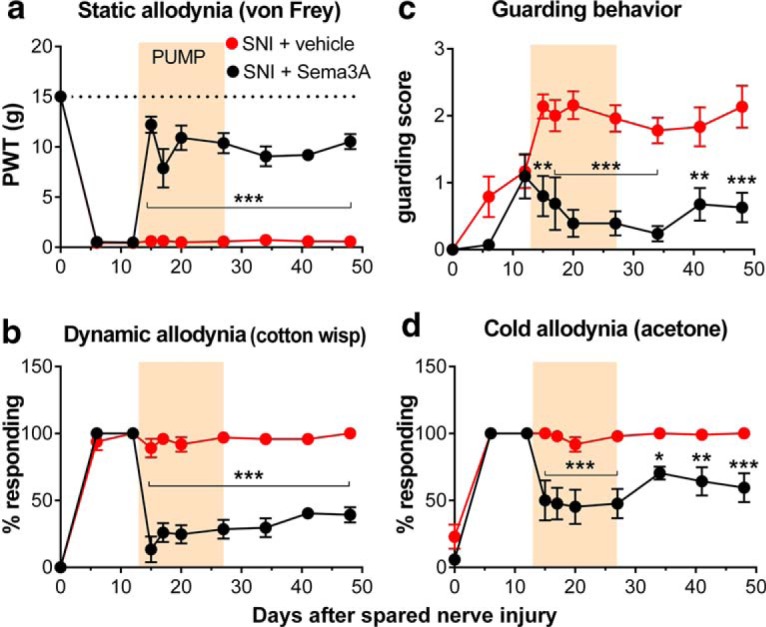
Applying semaphorin 3A to the injury site reduces established pain in the spared nerve injury model. Averaged baseline behaviors are plotted on day 0. Starting on day 13, an osmotic pump added during a second surgery was used to locally perfuse the injury site with semaphorin 3A or vehicle (ACSF) for 14 d, as indicated by the shaded bar. Behaviors were measured 1 d prior to and 2 d after the pump surgery in order to demonstrate that the behavioral effects were significant within 2 d. ***a***, Static allodynia (threshold for withdrawal responses to von Frey filaments; dotted line indicates baseline; *F*_(1,9)_ = 104.40). ***b***, Dynamic allodynia (withdrawal responses to light stroking with a fine cotton wisp; *F*_(1,9)_ = 103.40). ***c***, Guarding score (maximum possible value is 3; *F*_(1,9)_ = 42.49). ***d***, Cold allodynia (withdrawal responses to a drop of acetone; *F*_(1,9)_ = 33.03). *p < 0.05; **p < 0.01; ***p < 0.001, significant difference between the two groups at the indicated time points. *N* = 4 male and 4 female rats (vehicle group); *N* = 3 male and 4 female rats (semaphorin 3A group). PWT, paw withdrawal threshold.

To determine whether disrupting regeneration could reverse established pain in the SNI model, we next applied semaphorin 3A to the neuroma starting after pain was well established. As shown in Figure 6, applying semaphorin 3A for 2 weeks, starting 2 weeks after the initial spared nerve injury, caused a rapid and sustained reduction of pain behaviors that outlasted the duration of the drug treatment. It should be noted that in order to install the tubing around the injury site at week 2, it was necessary to dissect away the surrounding thickened tissue and resect and remove the neuroma with tangled regenerating fibers distal to the ligature, so that the drug had access to the nerve endings. As shown by the data for the rats receiving vehicle, this resection did not per se reduce the pain behaviors.

## Discussion

Our results suggest that the active regeneration process per se may drive the persistence of neuropathic pain after a peripheral nerve is injured. We observed that suppressing the regeneration process reduced pain behaviors in two different pain models, the SNL model, in which effective regeneration resulting in some reinnervation of the peripheral targets was observed, and the longer-lasting SNI model, in which the regeneration process led to the formation of a neuroma rather than terminating with successful target reinnervation. In the SNL model, we found that *in vivo* perfusion of the ligated spinal nerve with the inhibitory axonal guidance molecule semaphorin 3A, starting at the time of spinal nerve ligation, greatly reduced mechanical pain behaviors observed in that model. There was no effect on the first day after surgery, but the effect was significant by day 5 and, importantly, lasted the duration of the experiment (8 weeks) although the pumps used lasted only 2 weeks. Although semaphorin 3A can also have effects on immune cells (Vadasz and Toubi, 2014), our experiments showing a similar effect using a completely different approach to blocking regeneration, GAP43 knockdown within the L5 DRG, provide further evidence that the key mechanism for reducing pain behaviors was interrupting nerve regeneration. Also consistent with a causal relationship between regeneration and pain, we observed that the much longer-lasting pain in the spared nerve injury model was (as first proposed by its originators) accompanied by a failure of effective reinnervation; instead, a neuroma formed at the injury site containing tangled fibers expressing the regeneration marker GAP43. Even established (day 14) pain behaviors in this model could be mitigated by local perfusion of semaphorin 3A into the injury site but not by resection of the neuroma per se. As in the spinal nerve ligation model, the semaphorin 3A effect long outlasted the duration of drug application. Hence, in several different experiments using different methods to disrupt regeneration, a temporary disruption of the regeneration process produced a very long-lasting reduction in pain behaviors.

In the spinal nerve ligation model, we found that the regenerating nerve was a major source of abnormal spontaneous activity in the proximal dorsal root fibers; this activity was also reduced by semaphorin 3A or by tetrodotoxin, effects lasting long after the period of drug application. These results suggest that spontaneous activity is closely associated with the active regeneration process, although further study is needed to elucidate mechanisms and causal direction. In regard to the role of spontaneous activity, there seems to be a disconnect between those who study peripheral regeneration and those who study pain. In the pain field, abnormal spontaneous activity in peripheral sensory neurons is well established as an important contributor to the development of pain behaviors in a variety of models, and blocking this activity blocks the development of pain (Devor, 2009). Although there is an extensive literature regarding the role of spontaneous activity in the growth of axons during development (Spitzer, 2006), there is apparently little work on its role in the regeneration of peripheral nerve in adults. Instead, much of the literature on spontaneous activity in the field of adult peripheral nerve regeneration is concerned with the phenomenon of enhanced initiation of regeneration (though not increased rate) following a very brief (e.g., 1 h) stimulation of the peripheral nerve immediately after nerve transection (Shapira and Midha, 2015). One article commonly cited on this topic (Enes et al., 2010) even proposes that the lack of activity in severed sensory neurons is the signal that initiates their regeneration program. However, this study relied heavily on *in vitro* studies of axon growth in cultured sensory neurons and *in vivo* fiber recordings that did not specifically quantify spontaneous activity types, and the authors did not reference the literature on early spontaneous activity in pain models.

Studies of peripheral nerve regeneration also emphasize the complex shift from a gene expression program centered on neurotransmission to the expression of regeneration-activated genes (RAGs). Some of these, including GAP43 and transcription factors such as SOX 11, have been shown to be required for regeneration (Huebner and Strittmatter, 2009; Jankowski et al., 2009). RAGs are activated by peripheral but not dorsal root axon transection, but prior conditioning by peripheral transection can stimulate the normally ineffective centrally directed regeneration response after a second dorsal root injury (Gordon, 2016). Proposed mechanisms for transmitting the information that the periphery has been injured and for activating the RAG program include increased cAMP, axonal transport mechanisms, calcium signaling, and peripherally derived growth factors.

Another unexpected finding of our study was that regeneration and functional reinnervation of the ligated and cut spinal nerve can occur in the spinal nerve ligation model. In studies using this model, the discussion almost invariably assumes that regeneration and/or reinnervation does not occur. Our study shows that this assumption is not necessarily correct; we observed the regeneration of fibers reaching as far as the hindpaw region within 4 weeks of initial injury, and these fibers were able to conduct action potentials and mediate responses to mechanical stimulation of distal receptive fields. The regenerated nerve was visible *in situ*, although experienced experimenters in our laboratory used the model for many years without noticing this. We therefore think that it is likely that regeneration and reinnervation also occur in the model as implemented in other laboratories. However, this needs to be confirmed; it is possible that differences in implementation may mean that regeneration is not observed in this model in every laboratory. Users of this model also generally assume that none of the behaviors are mediated by L5. In our study, von Frey responses were evoked by stimuli in the paw region that are normally innervated primarily by the intact L4 spinal nerve, but it is possible that L5-mediated behaviors may be observed in some studies, especially at later time points, depending on the details of the experimental protocol.

Our ability to see regenerated fibers reaching the hindpaw within 4 weeks after spinal nerve ligation (a distance of ∼100 mm) is consistent with the upper estimate of regeneration rates of ∼4 mm/d reported in rat studies of sciatic nerve transection models used to study peripheral regeneration (Savastano et al., 2014). More rapid recovery of proximal receptive fields compared with distal fields presumably reflects the shorter distance the fibers needed to grow. Although we observed the functionality of regenerated fibers, this does not prove that fibers precisely reinnervated their original targets and restored completely normal function; indeed, studies examining functional reinnervation in finer detail suggest that regenerated fibers do not completely normalize function (He and Jin, 2016). In addition, our methods were not suitable for evaluating cell loss, which has also been described in this model (Vestergaard et al., 1997; Tandrup, 2004), although it was clear in the fiber-recording experiments that many more fibers in a standard diameter filament could be activated in normal rats than after spinal nerve ligation, even 10 weeks after spinal nerve ligation. In addition, we did not conduct thermal testing and hence would not have observed the failure of many C fiber neurons to regenerate. We assume that the inability of the regenerating nerve to ever reinnervate distal mechanical receptive fields after the initial 14 d semaphorin 3A treatment is likely related to the lost capability of the degenerating distal nerve segment to provide the structural pathway for the regenerating fibers, due to the delay. Studies on the role of processes occurring in the distal segment in peripheral nerve regeneration demonstrate a limited time window over which regeneration can be supported (Mar et al., 2014; DeFrancesco-Lisowitz et al., 2015; Jessen et al., 2015).

In conclusion, our study provides evidence for a close association between peripheral nerve regeneration, generation of spontaneous activity, and pain. It is important for future studies to bridge the gaps between these different fields of study. Studies of peripheral regeneration need to consider whether improving regeneration also increases pain behaviors, while studies of methods to block chronic pain need to consider whether this comes at the cost of increased neuronal cell death or failure to achieve regeneration that was otherwise possible. For pain conditions such as phantom limb pain, where regeneration is not possible, or conditions where the window of opportunity for peripheral nerve regeneration has passed, our results in the spared nerve injury model suggest that surgical removal of the neuroma alone would not alleviate pain; but interrupting the regeneration program, even after pain is well established, may provide pain relief that long outlasts the application of the regeneration-blocking agent.

## Synthesis

Reviewing Editor: Mary Heinricher, Oregon Health & Science University Decisions are customarily a result of the Reviewing Editor and the peer reviewers coming together and discussing their recommendations until a consensus is reached. When revisions are invited, a fact-based synthesis statement explaining their decision and outlining what is needed to prepare a revision will be listed below.

Repair and regeneration of injured nerve tissue is frequently held up as the holy grail of research focused on nervous system injury and repair. But regeneration may come at significant cost. In this intriguing report, Xie and colleagues provide evidence that, at least for peripheral nerve injury, some aspects of regeneration also contribute to neuropathic pain. The authors were able to demonstrate unexpected functional reinnervation of distal structures following an experimental ligation of the L5 spinal nerve, and show that local application of semaphorin 3A (an inhibitory axon guidance molecule) or tetrodotoxin (to suppress neuronal activity) and siRNA knockdown of GAP43 (associated with axonal growth) reduce behavioral signs of neuropathic pain. Although there was no evidence of peripheral reinnervation in a second nerve injury model (spared nerve injury), application of semaphorin 3A to the neuroma which forms after this injury reduced abnormal pain behaviors. Taken as a whole, this work highlights the need to consider costs as well as benefits of enhancing regeneration of injured nerve.

The manuscript is significant, and represents a new perspective on pain following nerve injury. The methods used are appropriate, and necessary controls are in place, including a very nice experiment showing that re-transection of the regrown spinal nerve eliminated evoked activity. There are nonetheless two important concerns.

Statistical analysis. The journal (and I) require that the relevant statistical parameter, degrees of freedom (when relevant) p-value and n be provided for each analysis (at least the overall analysis, results of post-hoc tests can be provided as p-value only, either in the text or indicated in the figures). A specific concern relates to pseudo-replication - what is the actual n for the electrophysiological experiments? The different “filaments” are not independent, and cannot be employed in the analysis as if they were. These data should be provided as averaged (or summed) number of active fibers in each animal. One suspects that the authors have considered the issue of pseudo-replication given the material on p. 15 where they state that the same outcome was obtained using the more appropriate approach, which is the average value for each animal.

The paper could be better written. Although not incorrect in grammar or syntax, the arguments are not well-articulated, and could easily lead a reader to be confused about the point of the paper (such as which afferent fiber population was responsible for activating the afferents). I have made some comments on an attached pdf file, but strongly encourage the authors to have a reader not intimately familiar with the work give them a thorough review. As is, it requires a dedicated reader to understand what is interesting and important about the findings.
